# Supplementary material for: Compositional Changes in Hydroponically Cultivated Salicornia europaea at Different Growth Stages
Source: Plants (Basel). 2023 Jun 28;12(13):2472. doi: 10.3390/plants12132472 (PMC10346760; doi:10.3390/plants12132472)
Supplement: Supplementary file 1 [file plants-12-02472-s001.zip › plants-2433693-supplementary.pdf]

Compositional Changes in Hydroponically Cultivated *Salicornia europaea* at Different Growth Stages

Ariel E. Turcios [1,†], Lukas Braem [2,†], Camille Jonard [2], Tom Lemans [2], Iwona Cybulska [2], Jutta Papenbrock [1,*]

Supplementary material

Table S1. Correlation analysis of the different growth parameters and organic compounds in *Salicornia europaea*

	Week	*Ash*	*Lignin*	*Glucose*	*Xylose*	*Arabinose*	*Insoluble_Compounds*	*Water_Extractives*	*Ethanol_Extractives*	*Volatile_Loss*	*Residues*	*Protein*	*Aboveground Fresh Biomass (g)*	*Aboveground Dry Biomass (g)*	*Water_Biomass (%)*
Week	1														
Ash	-0.40098	1													
Lignin	0.851591	-0.14737	1												
Glucose	0.835518	-0.27713	0.757065	1											
Xylose	0.791573	-0.2018	0.714753	0.972595	1										
Arabinose	0.308672	-0.15112	0.106448	0.245973	0.414876	1									
Insoluble_Compounds	0.500288	-0.704	0.258368	0.239173	0.259451	0.550436603	1								
Water_Extractives	-0.82519	0.36582	-0.75496	-0.70216	-0.72979	-0.554077052	-0.703204583	1							
Ethanol_Extractives	-0.10873	-0.01162	0.029717	-0.16009	-0.08044	0.293949158	0.352004859	-0.425664153	1						
Volatile_Loss	0.689606	-0.36904	0.551801	0.307722	0.262783	0.291395898	0.653001355	-0.774200255	0.402946182	1					
Residues	0.899788	-0.36164	0.805883	0.86839	0.892322	0.5394323	0.608427329	-0.933820045	0.119615137	0.582860349	1				
Protein	-0.81452	0.041975	-0.75617	-0.78665	-0.77006	-0.254488427	-0.199813185	0.55114507	0.439523918	-0.288207854	-0.7662688	1			
Aboveground Fresh Biomass (g)	0.943649	-0.23654	0.871033	0.800404	0.740402	0.190806139	0.334764491	-0.720361941	-0.216349945	0.614531723	0.81985397	-0.89815	1		
Aboveground Dry Biomass (g)	0.982312	-0.45829	0.801542	0.855152	0.816865	0.335548031	0.538717119	-0.850019053	-0.032711667	0.683993209	0.90974431	-0.75717	0.905450489	1	
Water_Biomass (%)	-0.9233	0.519243	-0.70019	-0.81748	-0.79333	-0.350491614	-0.594393711	0.819997953	-0.001061885	-0.616044106	-0.8802014	0.649578	-0.769558299	-0.959749693	1

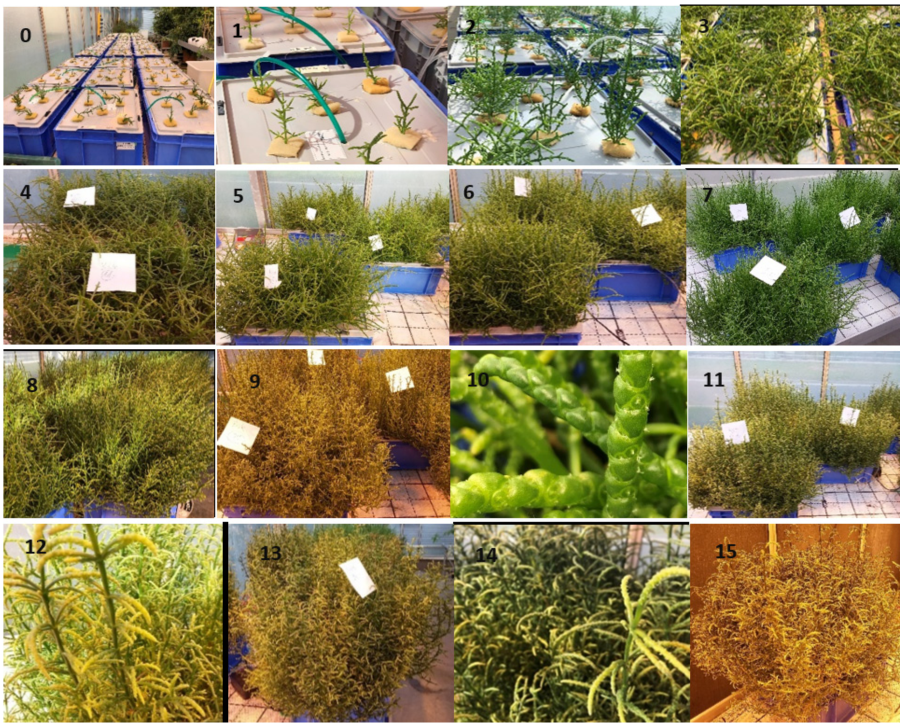

Figure S1. Growth and development of *Salicornia europaea* plants during 15 weeks after transplanting under hydroponic conditions. Numbers represent the number of weeks after transplanting.
